# Supplementary material for: Transformer brain encoders explain human high-level visual responses
Source: ArXiv. 2026 Feb 5:arXiv:2505.17329v3. Preprint. [Version 3] (PMC12889847)
Supplement: Supplement 1 [file NIHPP2505.17329v3-supplement-1.pdf]

A Supplementary Material

A.1 Encoding accuracies for Subjects 2, 3 and 7

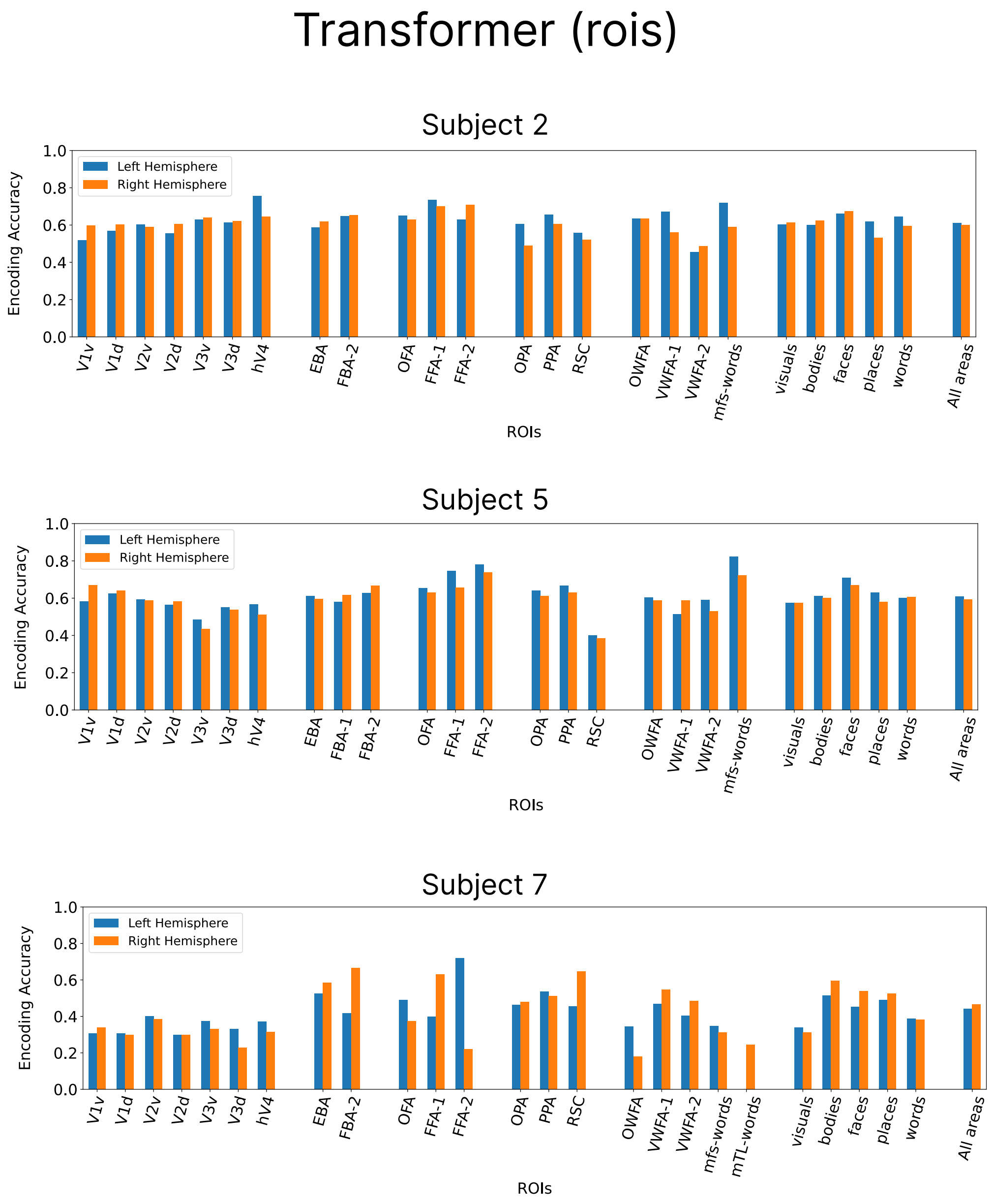

Figure S1: Encoding accuracy (fraction of explained variance) shown for Subjects 2, 5, and 7 for individual ROIs and for ROI clusters for the two hemispheres. The transformer model uses ROIs for decoder queries and features from the last layer of the DINOv2 backbone.

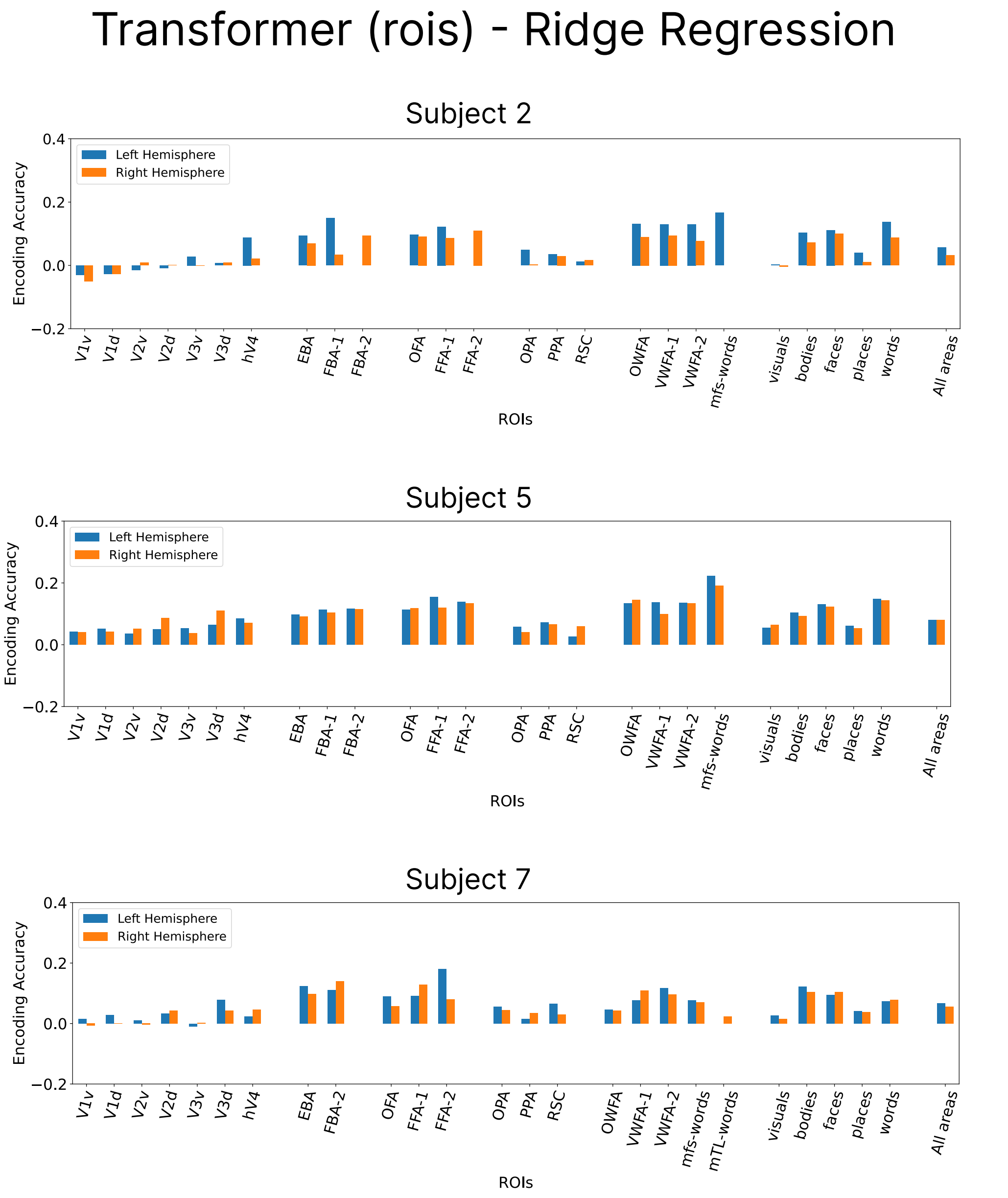

Figure S2: The differences in encoding accuracy between the transformer and the ridge regression models shows that the transformer encoder better predicts especially higher visual areas.

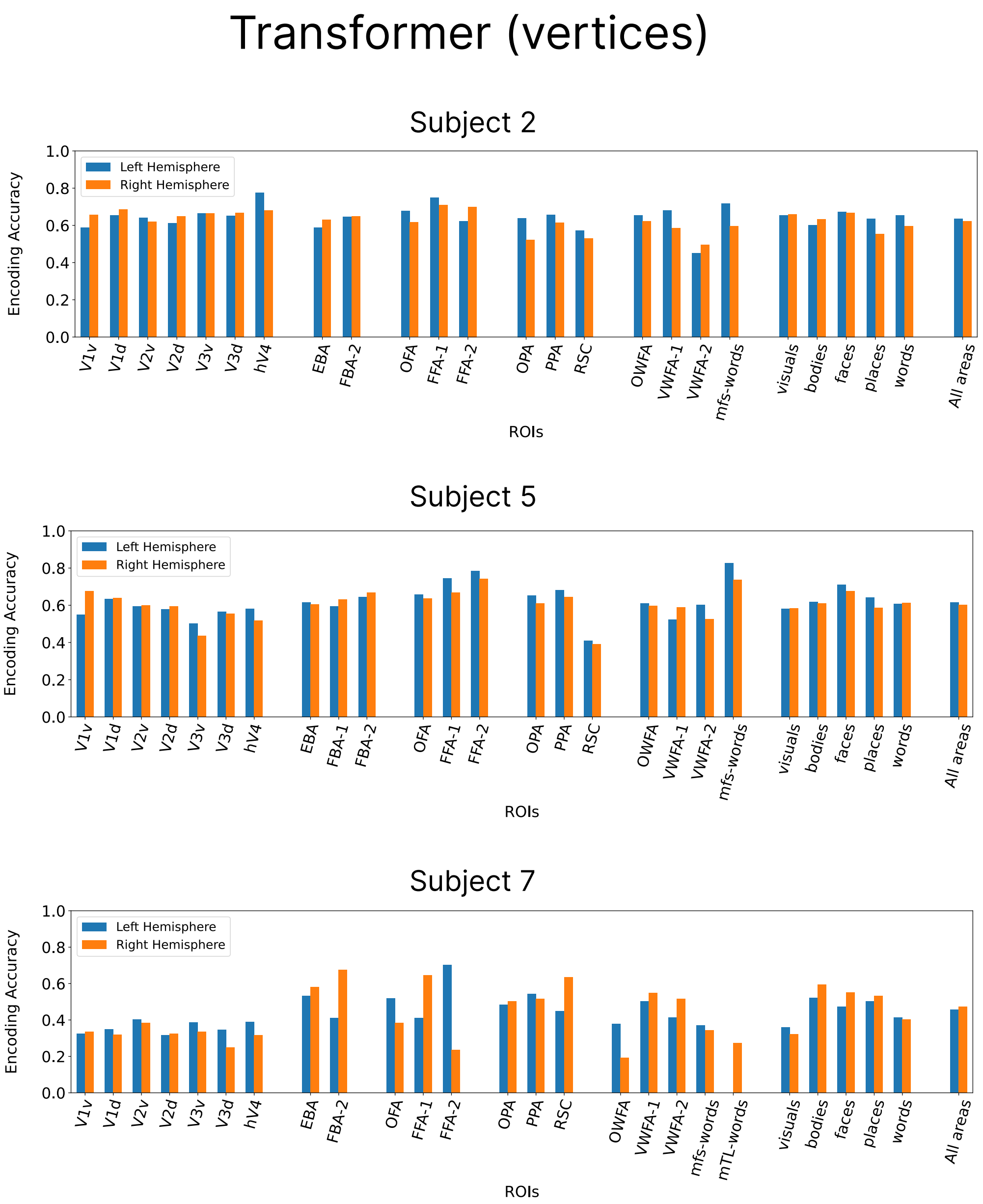

Figure S3: Encoding accuracy (fraction of explained variance) shown for Subjects 2, 5, and 7 for individual ROIs and for ROI clusters for the two hemispheres. The transformer model uses vertices for decoder queries and features from the last layer of the DINOv2 backbone.

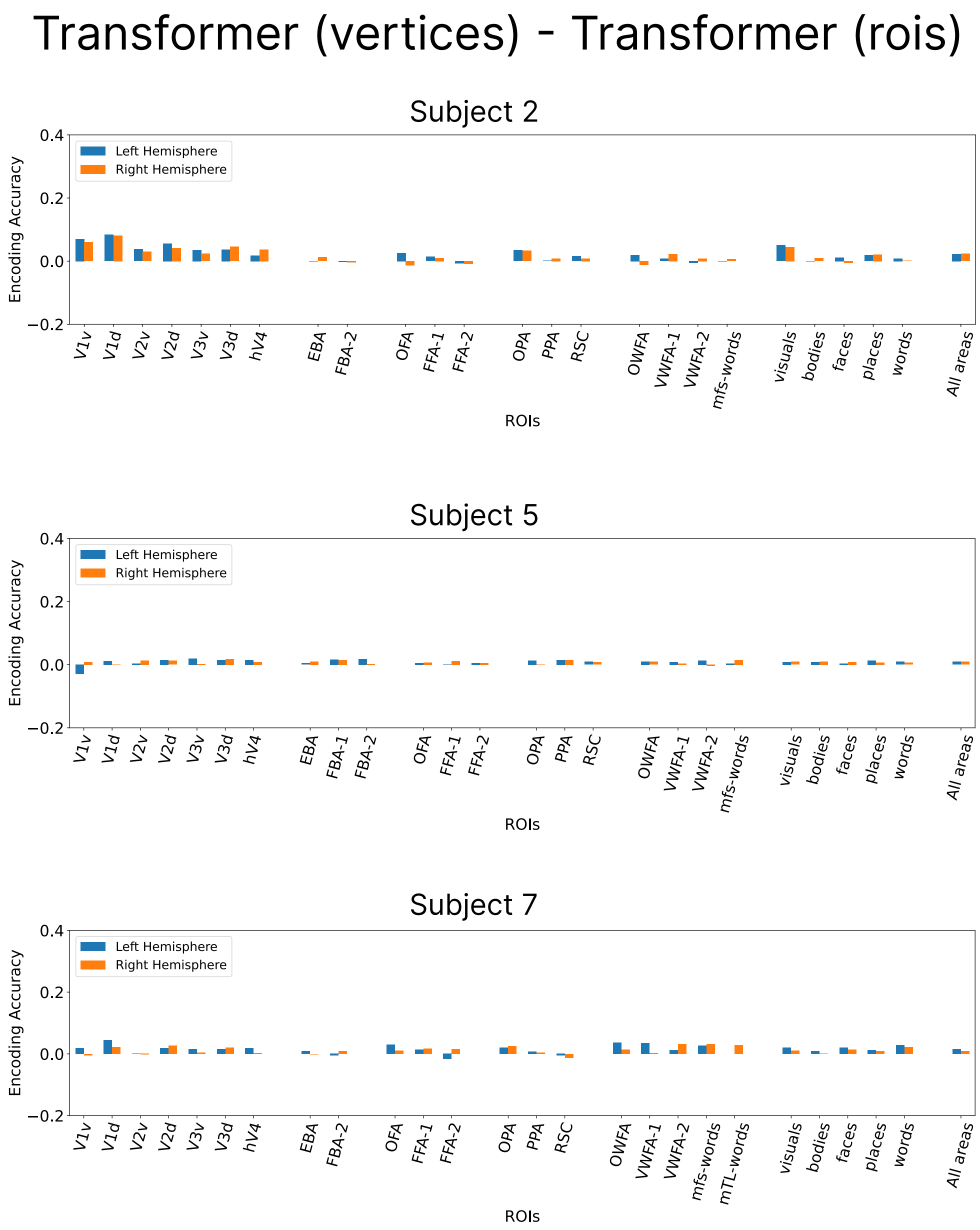

Figure S4: The differences in encoding accuracy between the transformer model using vertices and the model using ROIs as decoder queries. The figure shows that any potential improvement in the former is driven by better prediction of early visual areas.

A.2 Category selectivity of attention maps

To quantify the category selectivity of attention maps, we classified each pixel of the test set images using YOLOv5 [22] and YOLOv8-face [11] into five categories: background, face, body (pixels classified as person but not as face), animal, and food. For each ROI, we calculated and resized its attention maps to 434×434 for these images, and reported the categories of the 2k pixels with top attention values. We found that the category selectivity is consistent with ROI labels, with EBA most selective for body, FFA most selective for face, and OPA/PPA/RSC most selective for background.

Table 7: Category selectivity of ROI attention for subject 1.

	background	face	body	animal	food
EBA	0.03	0.36	0.61	0.00	0.00
FFA-1	0.00	0.79	0.16	0.05	0.00
FFA-2	0.00	0.83	0.17	0.00	0.00
OPA	0.54	0.12	0.14	0.05	0.15
PPA	0.44	0.25	0.11	0.10	0.10
RSC	0.66	0.23	0.11	0.00	0.00

Table 8: Category selectivity of ROI attention for subject 2.

	background	face	body	animal	food
EBA	0.03	0.25	0.72	0.00	0.00
FFA-1	0.05	0.57	0.19	0.18	0.00
FFA-2	0.05	0.53	0.27	0.15	0.00
OPA	0.74	0.15	0.06	0.05	0.00
PPA	0.78	0.11	0.08	0.03	0.00
RSC	0.71	0.19	0.09	0.00	0.00

Table 9: Category selectivity of ROI attention for subject 5.

	background	face	body	animal	food
EBA	0.29	0.26	0.40	0.05	0.00
FFA-1	0.12	0.67	0.13	0.08	0.00
FFA-2	0.10	0.59	0.28	0.03	0.00
OPA	0.31	0.33	0.20	0.16	0.00
PPA	0.36	0.29	0.20	0.15	0.00
RSC	0.08	0.37	0.50	0.00	0.06

Table 10: Category selectivity of ROI attention for subject 7.

	background	face	body	animal	food
EBA	0.38	0.05	0.43	0.09	0.05
FFA-1	0.00	0.88	0.02	0.10	0.00
FFA-2	0.17	0.20	0.38	0.20	0.05
OPA	0.40	0.18	0.14	0.28	0.00
PPA	0.51	0.29	0.16	0.05	0.00
RSC	0.56	0.25	0.17	0.02	0.00

A.3 Analyzing learned ROI queries

We analyzed the representational similarity of learned ROI queries, and report the average cosine similarity between each pair of ROIs across 20 models trained using five different random seeds and four different DINOV2 backbone layers in Figures S5, S6, S7, S8. These figures show the visual and semantic similarity between the ROIs as reflected in the learned queries for the subjects. We observed that ROIs with shared category selectivity form clusters (faces, places, bodies, or words) in the similarity matrix, exhibiting greater representational similarity within each category type.

We also see a clear divide between categorical and non-categorical areas. Additionally, ROIs within the ventral early visual areas (V1v, V2v, V3v) are more similar to one another than to their dorsal counterparts (V1d, V2d, V3d), and vice versa (the checkerboard patterns), reflecting the anatomical and functional organization of the visual cortex, and that the attention will be mostly driven by spatial information.

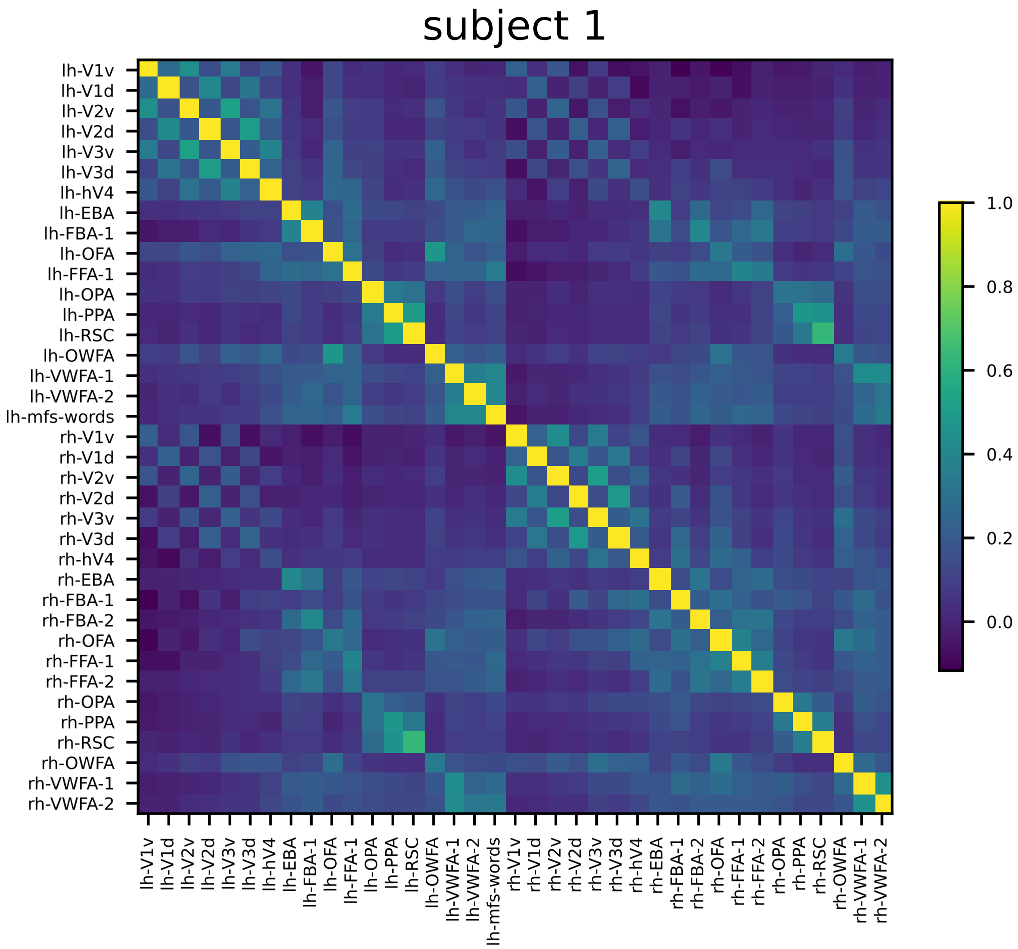

Figure S5: Cosine similarity between learned ROI queries for subject 1. Each entry in the matrix represents the average cosine similarity between the query for the ROI indicated by the row label and that indicated by the column label. ROIs from the left hemisphere are labeled with ‘lh’, and those from the right hemisphere with ‘rh’. Results are averaged across 20 models, trained using five random seeds and four different backbone layers.

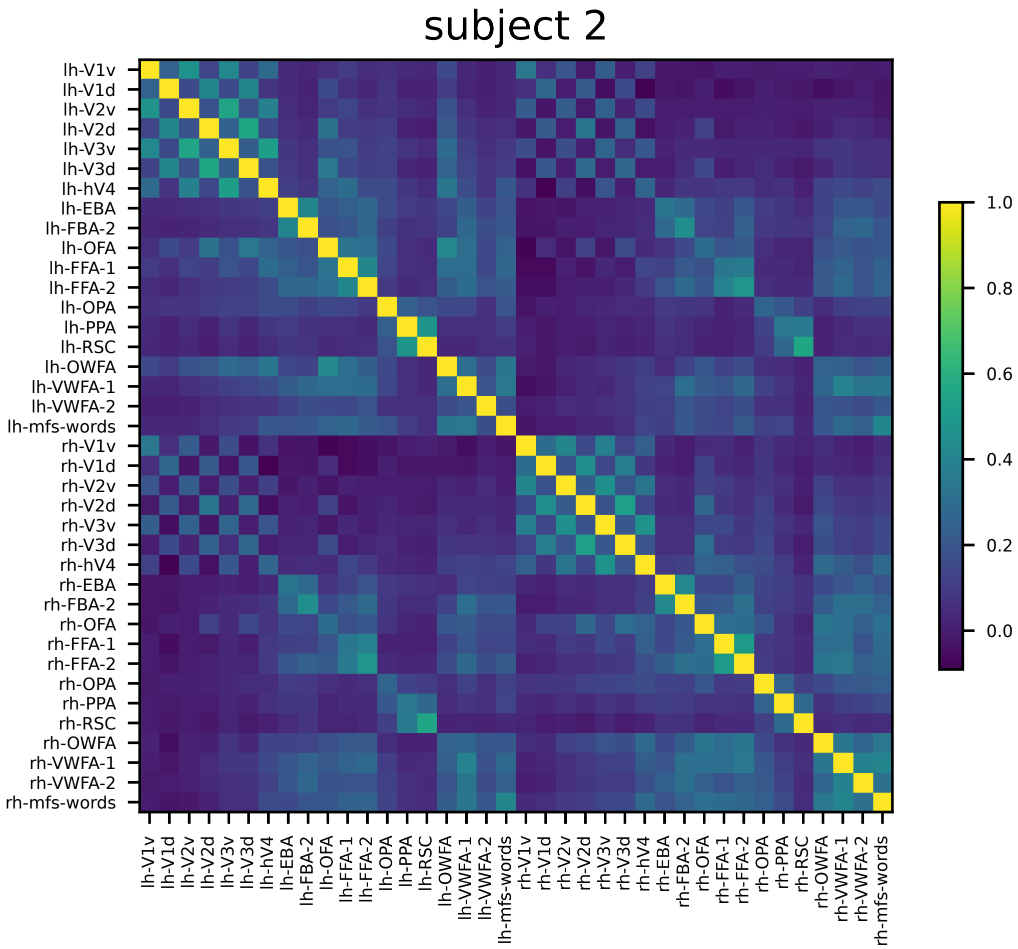

Figure S6: Cosine similarity between learned ROI queries for subject 2.

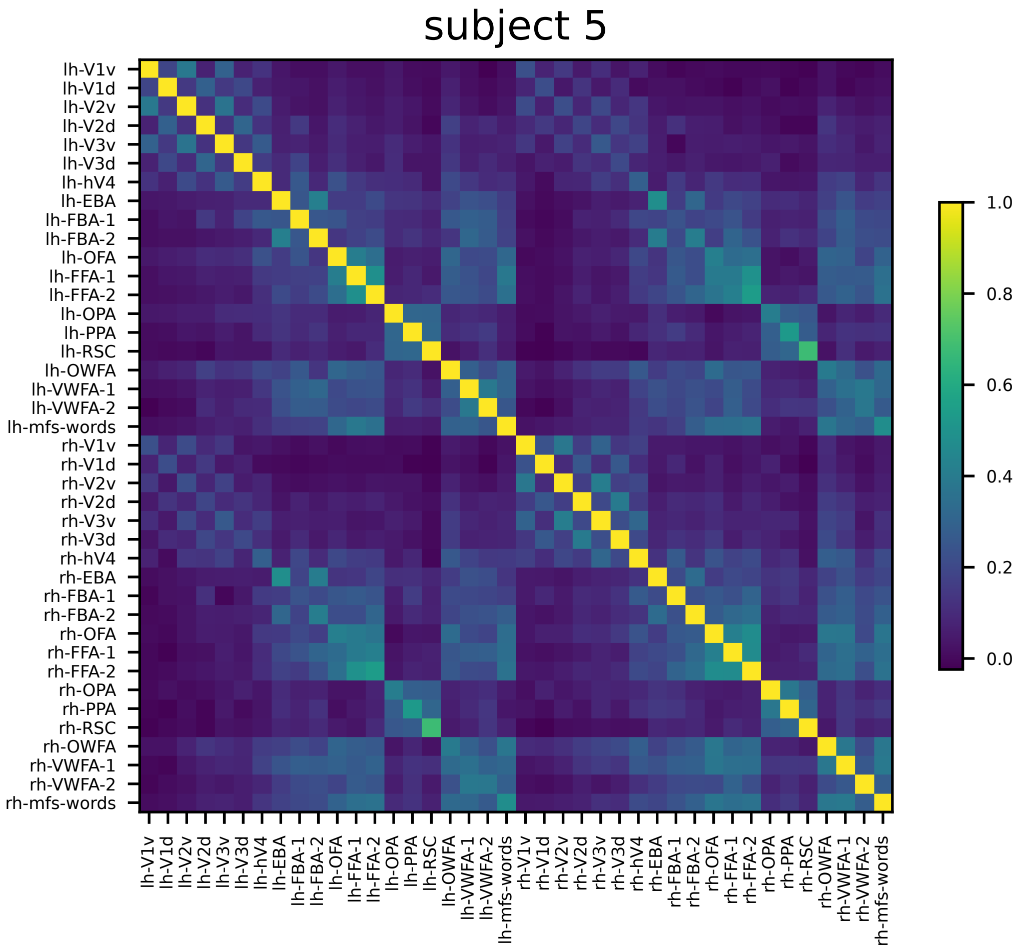

Figure S7: Cosine similarity between learned ROI queries for subject 5.

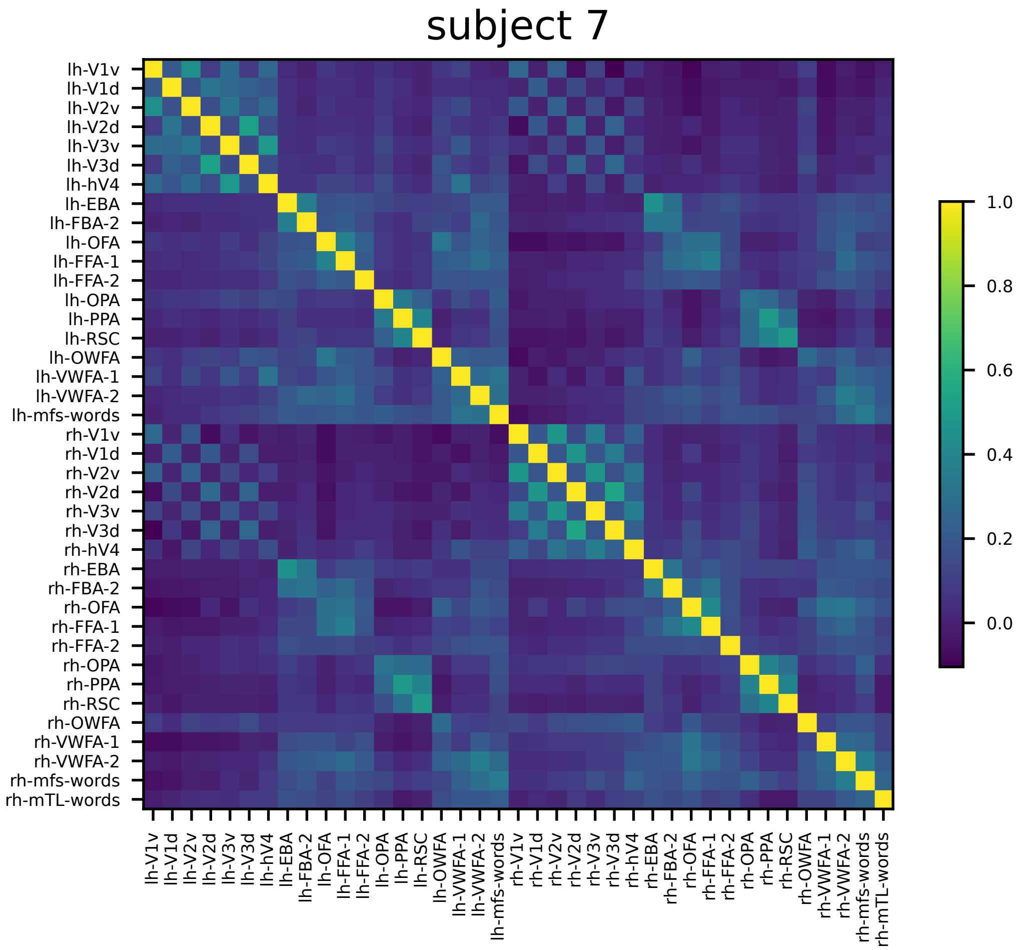

Figure S8: Cosine similarity between learned ROI queries for subject 7.

A.4 Generating maximally activating images for ROIs

BrainDiVE [32] is a generative framework for synthesizing images predicted to activate specific regions of the human visual cortex. It guides the denoising steps of a diffusion model using gradients derived from a brain encoding model. Given the strong performance of our encoding model in predicting brain activity, we tested whether it could also effectively guide image generation within the BrainDiVE framework. We generated 200 images optimized to maximally activate the average predicted response of a specific ROI cluster, and display the top five in Figure S9, S10. The categories of the generated images are consistent with the reported category selectivity of each ROI cluster in the literature.

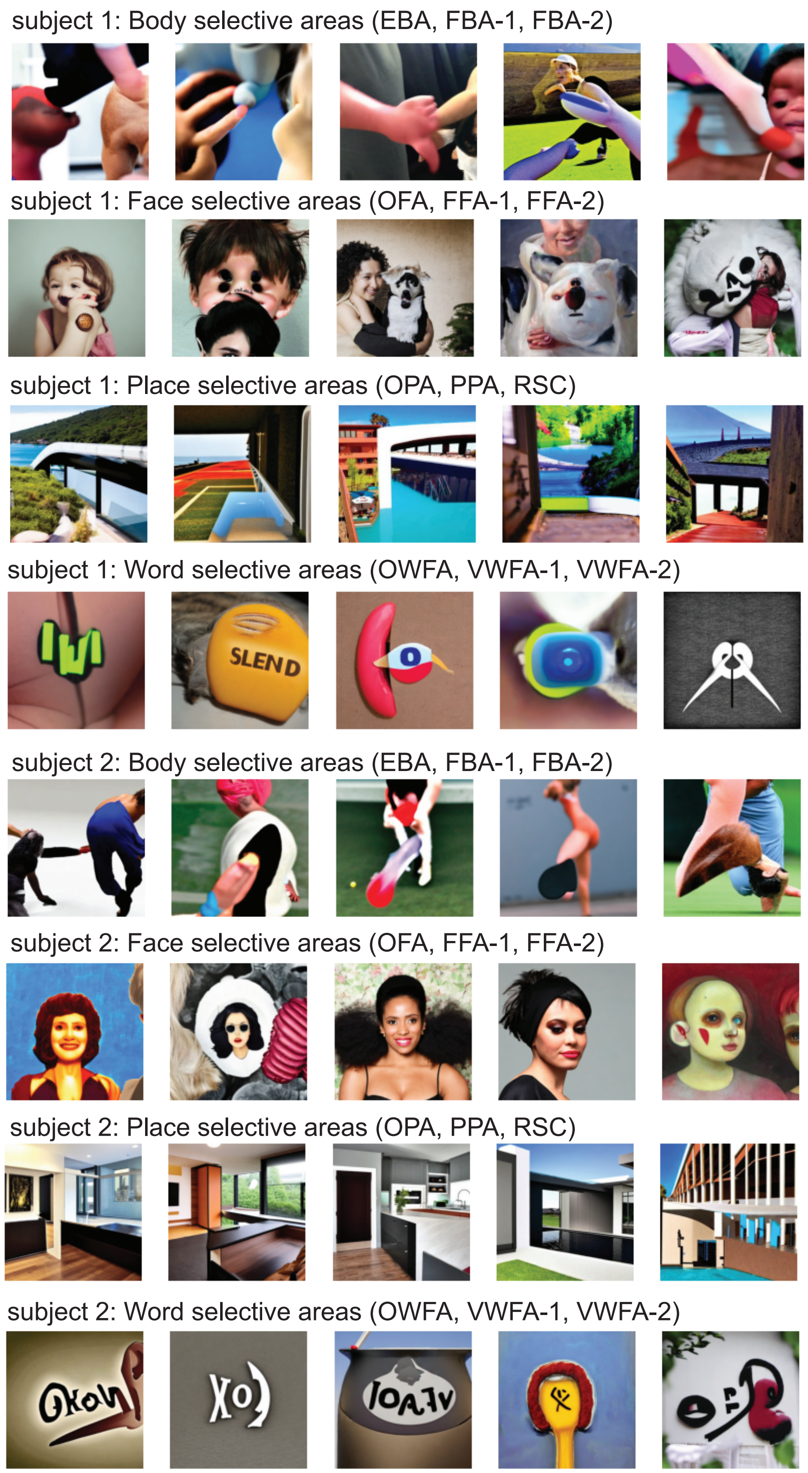

Figure S9: Images generated to maximally activate different ROI clusters for subjects 1 and 2. Using our encoding model within the BrainDiVE framework, we generated 200 images predicted to maximally activate a specific ROI cluster for a given subject (indicated by the row titles). For each cluster, we display the top five images with the highest predicted activation, as determined by our encoding model.

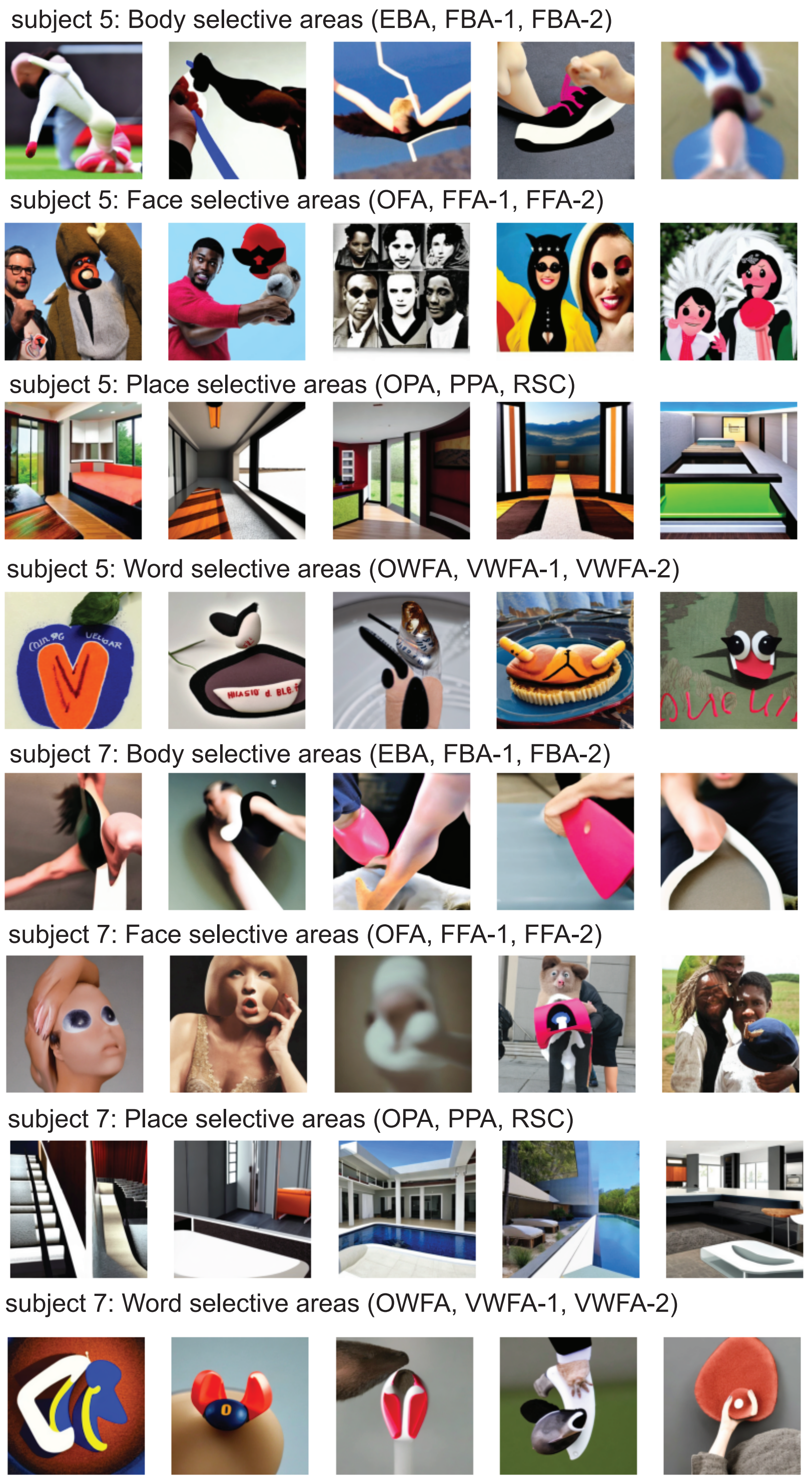

Figure S10: Images generated to maximally activate different ROI clusters for subjects 5 and 7

B Compute used

We used GPUs (NVIDIA L40s), memory, and storage resources from an internal cluster. Storage for the entire project totals roughly 3TB. Training the model used roughly 4,000 GPU hours. Running the remaining experiments used roughly 1,000 GPU hours. The full project required more compute than these estimates due to failed experiments, experiments not included in the paper, and model iteration.
